# Supplementary figures and images for: A targeted isotope dilution mass spectrometry assay for osteopontin quantification in plasma of metastatic breast cancer patients
Source: PLoS One. 2023 Jun 29;18(6):e0281491. doi: 10.1371/journal.pone.0281491 (PMC10309610; doi:10.1371/journal.pone.0281491)

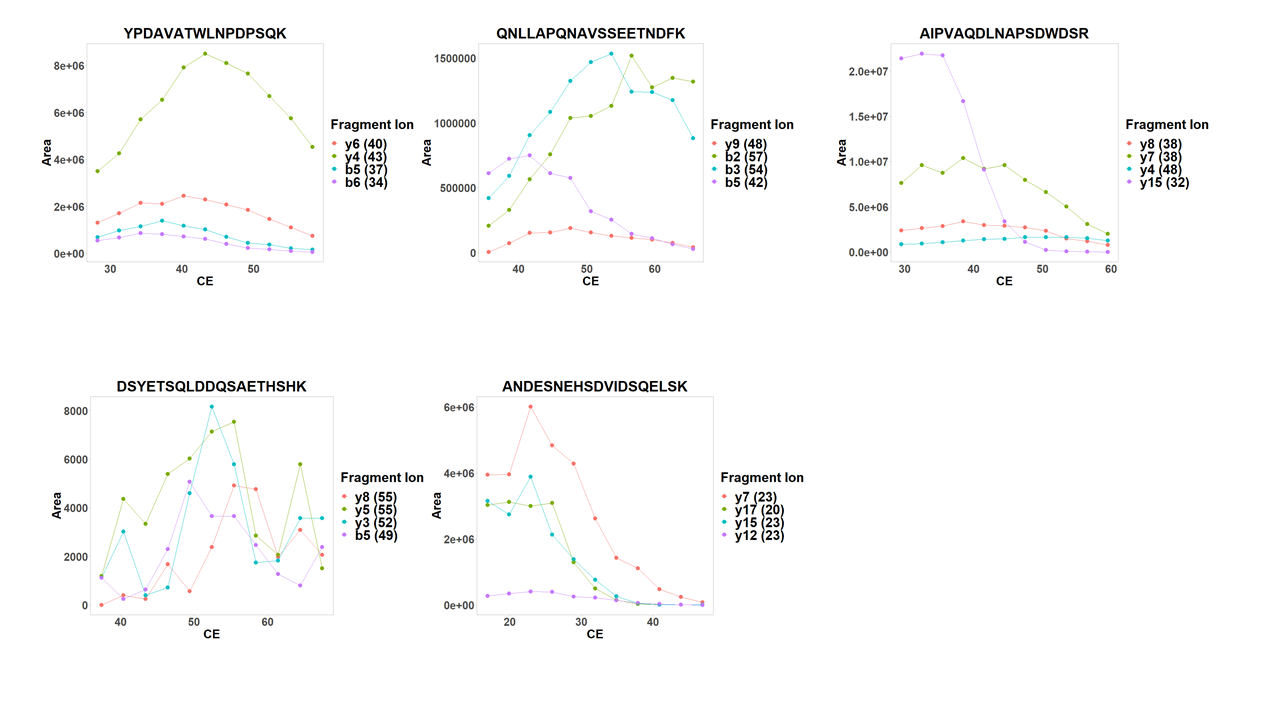

Supplement: S1 Fig — Optimization of collision energy for five target OPN tryptic peptides. A 200 ng of tryptic digest of OPN protein was injected for parameter optimization. (TIF) [file pone.0281491.s001.tif]
