# Supplementary material for: A targeted isotope dilution mass spectrometry assay for osteopontin quantification in plasma of metastatic breast cancer patients
Source: PLoS One. 2023 Jun 29;18(6):e0281491. doi: 10.1371/journal.pone.0281491 (PMC10309610; doi:10.1371/journal.pone.0281491)
Supplement: Attachment — Submitted filename: Response to Reviewers.docx [file pone.0281491.s003.docx]

Response to Reviewers

We thank the reviewers for the comments and have accepted their comments and made the requested changes as described below (blue text).

6. Review Comments to the Author

Please use the space provided to explain your answers to the questions above. You may also include additional comments for the author, including concerns about dual publication, research ethics, or publication ethics. (Please upload your review as an attachment if it exceeds 20,000 characters)

Reviewer #1: The authors have diligently addressed all the raised concerns. The manuscript after revision has significantly improved.
In the Supplemental Figure S1 – Collision Energy Optimization curves, the axes labels are not legible. It is advised to increase the font size for better readability.

Figure S1 has been revised to increase the font size.

Reviewer #2: The authors developed an OPN quantifcation methodology that does not require use of antibodies and with a simple sample preparation procedure with LOD at 39 ng/mL, which is a good result. However, the assay has got insufficient sensitivity to quantify OPN in the samples from some breast cancer patients, as the detected concentrations were between 17 and 53 ng/mL. Therefore, I think it would be fair to the potential reader to state also in the abstract: (1) OPN levels detected in the pilot study plasma samles, and, (2) that the assay is useful rather for plasma from patients of high grade tumors, where the expected OPN concentration is higher.

We thank the reviewer for this comment and have added the following text to the abstract as requested: The assay was applied to the analysis of plasma OPN in metastatic breast cancer patients, where levels from 17 to 53 ng/mL were detected. The sensitivity of the method is higher than previously published methods and sufficient for OPN detection in large, high grade tumors but still requires improvement in sensitivity to be widely applicable.

Reviewer #3: The manuscript has been considerably improved by addressing all comments satisfactorily. It will be very useful to readers and is now ready for publication.
